# Counting mitoses in gastrointestinal stromal tumours (GISTs): variable practices in the real-world setting and their clinical implications

**DOI:** 10.1007/s00428-022-03454-w

**Published:** 2022-11-23

**Authors:** Michela Campora, Michele Paudice, Alessandro Gambella, Danila Comandini, Paola Parente, Marta Sbaraglia, Angelo Paolo Dei Tos, Federica Grillo, Luca Mastracci

**Affiliations:** 1 Anatomic Pathology, Ospedale Santa Chiara, Trento, Italy; 2 IRCCS Ospedale Policlinico San Martino, Largo Benzi 10, 16132 Genoa, Italy; 3 Pathology Unit, Department of Surgical Sciences and Integrated Diagnostics (DISC), University of Genoa, Genoa, Italy; 4 Department of Medical Sciences, University of Turin, Turin, Italy; 5 Oncology Unit, Department of Oncology and Haematology, IRCCS Ospedale Policlinico San Martino, Genoa, Italy; 6 Fondazione IRCCS Casa Sollievo della Sofferenza, Pathology Unit, San Giovanni Rotondo (FG), Italy; 7 Department of Pathology, Azienda Ospedale-Università Padova, Padua, Italy; 8 Department of Medicine (DIMED), University of Padua School of Medicine, Padua, Italy

**Keywords:** Gastrointestinal stromal tumour, Prognosis, Mitotic count, Risk stratification

## Abstract

Mitotic count (MC) is an important prognostic indicator in gastrointestinal stromal tumours (GISTs). Though MC evaluation was initially proposed in 50 HPFs, recent international guidelines recommend that MC be performed on 5 mm^2^ because HPFs may have different areas depending on the ocular field number (FN) of the utilized light microscope. Performing MC on different areas leads to a non-standardized evaluation and erroneous risk stratification. The aim of the study was to audit real-life MC practices with special emphasis on possible risk stratification errors. A survey was administered to Italian pathologists to evaluate the following: method used for MC (5 mm^2^ versus 50 HPF); FN of the light microscope; prognostic scheme for risk stratification. Based on the results of the survey, 100 GISTs (25/risk class using Miettinen prognostic scheme) were retrieved and MC performed using 5 mm^2^ versus the corresponding mm^2^ area sizes of 50 HPFs with variable FNs (18, 20, 22). The survey demonstrated that the majority of pathologists (64.5%) use 50 HPFs with various FNs leading to excessive area size. The most frequently used prognostic scheme is that by Miettinen. Using this prognostic scheme and counting mitoses in 5 mm^2^ versus 50 HPFs with FNs 18, 20 and 22, a change in risk class was identified ranging from 10 to 41%, depending on FN. In conclusion, this study demonstrates that MC is still frequently performed on 50 HPF, with area sizes exceeding the specified 5 mm^2^ by far.

## Introduction

Gastrointestinal stromal tumours (GISTs) are rare mesenchymal tumours originating in the digestive tract with variable prognosis. Mitotic count (MC) is one of the main prognostic factors, together with tumour size, tumour site and rupture of the tumour capsule. MC, as an indicator of aggressive behaviour, was initially proposed in 2002 [1] and its use was subsequently confirmed in more elaborate prognostic schemes [2, 3]. All the aforementioned studies have used MC as a *categorical* variable with precise cutoffs between prognostic classes while the use of MC as a *continuous* variable has been proposed in contour maps [4] and in different types of nomograms [5, 6]. In the literature, MC has been expressed as number of mitoses in 50 high-power fields (HPF), and only more recently, as number of mitoses in 5mm^2^.

Different factors can affect the reliability of MC: inter-/intra-observer variability, identification of ‘hot spots’, differential diagnosis with apoptotic bodies, pyknotic cells, karyorrhectic debris, pre-analytic factors and variable field sizes on different oculars/microscopes [7]. While it may prove difficult to uniform operator-dependent factors, standardization can be attempted for the latter two (pre-analytic factors and field size). In particular, HPF (×40 objective) size depends on the field number (FN) of the ×10 ocular which ranges between 18 and 26.5 mm, with 20–22 mm as the most frequently used FNs in new models of light microscopes [8].

Considering that for an HPF with an 18-mm FN, the area is considerably smaller (0.159 mm^2^) than an HPF with a 22-mm FN (0.237 mm^2^), recent guidelines [9–13] suggest that MC should be counted as the number of mitoses/total area of 5 mm^2^, thus bypassing the unstandardized HPF unit. This is an important attempt to uniform this information in histological reports as pathologists use microscopes with different oculars, different FNs and therefore different HPF areas.

Unfortunately, this suggestion does not provide details on how this new method can be adapted to the prognostic schemes (all based on 50 HPFs). The ESMO-EURACAN [9] guidelines state that the number of mitoses in 5mm^2^ replaces the former 50 HPFs — ‘The mitotic count has a prognostic value and should be expressed as the number of mitoses on a total area of 5 mm^2^ [which should replace, *and is equivalent to*, the 50 high-power field area, in order to avoid variability]’, while the Spanish Group for Sarcoma Research — GEIS — guidelines [10] state that ‘a total area of 5 mm^2^ *is equivalent to* the former 50 HPF’. Furthermore, TNM 2017 [11] suggests that ‘the mitotic rate of GIST is best expressed as the number of mitoses per HPF (total area 5 mm^2^ in 50 fields)’. These affirmations are confusing because, as underlined above, an area of 5 mm^2^ does not correspond simply to 50 HPFs [8].

Conversely the Asian Guidelines [12] state that ‘the actual size of a high-power field may differ between microscopes when obtaining mitotic counts’. Hence, the panels recommend that mitotic counts are expressed as per 5 mm^2^ fields while the French Intergroup Clinical Practice Guidelines [13] specifically state that ‘mitotic count is determined by Miettinen on a 5 mm^2^ surface, not on 50 HPF’.

The original studies by Miettinen et al. from 2005 [14] and 2006 [15] specified that *using their microscope* (make and FN not specified), 50 HPFs corresponded to an area of 5.3 mm^2^ while, on most modern microscopes, 5 mm^2^ corresponds to 20–25 HPFs [13]. On the basis of these two large studies on gastric and jejunum-ileum GISTs [14, 15], a risk classification, which is widely used even today, was proposed by the Armed Forces Institute of Pathology, which incorporates tumour size, tumour site and mitotic count expressed in 50 HPFs [2, 16].

In consideration of this variability in mitotic counting area in GISTs, our first aim was to verify how pathologists currently report MC in the real world and which are the most frequently used prognostic schemes. With this in mind, the second aim of this study was to verify the frequency of change of risk class, between using MC on 5 mm^2^ and MC in 50 HPFs using microscope oculars with different FNs (comprised between 18 and 22) based on the most frequently encountered FN used in practice in our survey.

## Materials and methods

### Survey

A brief survey was created and administered to Italian pathologists using two different methods: (1) mailing list of the Italian Group of Gastrointestinal Pathologists (GIPAD); (2) mailing list of the Italian Society of Diagnostic Anatomic Pathology and Cytology — Italian Division of International Academy of Pathology (SIAPEC-IAP). Participation in the survey was spontaneous and participants were completely anonymized.

The survey comprised 5 questions:

1. Field of interest: (a) gastrointestinal pathology and/or soft tissue pathology only; (b) other fields but including gastrointestinal and/or soft tissue pathology; and (c) other fields not including gastrointestinal and soft tissue pathology;
2. Years of professional activity (post training);
3. MC evaluation using (a) 5 mm^2^; (b) 50 HPF; and (c) other method (i.e. digital pathology);
4. Field number (FN) — ocular dimension of the regularly used light microscope: (a) 18; (b) 20; (c) 22; (d) 26; and (e) other (specify)
5. Prognostic scheme used in the pathology report: (a) Fletcher CD. Human Pathology 2002;33:459-465 [1]; (b) Miettinen M. Seminars in Diagnostic Pathology 2006;23:70-83 [2]; (c) Joensuu H. Human Pathology 2008;39:1411-1419 [3]; (d) Rossi S. et al., Am J Surg Pathol 2011;35:1646-1656 [5]; (e) Joensuu H et al., Lancet Oncol 2012;13:265-274 [4]; (f) Other (specify); and (g) none.

### Histologic evaluation

One hundred cases were retrieved from the Genoa GIST database on the basis of the prognostic scheme by Miettinen [2] in order to examine 25 very low, 25 low, 25 moderate and 25 high-risk GISTs. In all these cases, MC was expressed as number of mitoses/5 mm^2^.

The study was conducted according to the guidelines of the Declaration of Helsinki. Ethics committee approval was not required due to the following considerations: all data related to patients’ identification were anonymized; only original slides stained with haematoxylin and eosin were reassessed and no new sections were produced; this study has a speculative aim and results will not modify in any way the diagnosis, prognosis or add new clinical information useful for patient management.

All cases were simultaneously reassessed for MC, by two pathologists using a Leitz Laborlux D microscope (Leica Microsystems, Germany), with an 18-mm FN. MC was evaluated as mitoses/5 mm^2^ in order to confirm the original class of risk attribution.

Mitotic figures were counted as described by Miettinen et al. [14, 15]: recognition of the most mitotically active area (hot spot) using a 40× lens and counting continuously. For a FN of 18, MC was counted in 5 mm^2^ which corresponds to 31 HPFs. Count was than increased to 50 HPFs for 18 FNs which corresponds to an area of 8 mm^2^.

To make sure that the same hot spot area was analysed for each count (and not add further variability in field choice), it was decided that, rather than change microscope when evaluating 50 HPFs with different FNs, the number of HPFs would be increased to reach the corresponding area when 50 HPFs are evaluated using each FN. To recreate *50 HPFs at 20 FNs* (corresponding to 9.8 mm^2^), 61.5 HPFs were analysed. To recreate *50 HPFs at 22 FNs* (corresponding to 11.9 mm^2^), 74.5 HPFs were analysed. See Table 1.

**Table 1:** Main results from the survey, dividing participants who count mitoses in 5 mm^2^ versus in 50 HPF

Mitotic count	5 mm^2^							50 HPF						
Number of pathologists	39/110 (35.5%)							71/110 (64.5 %)						
Area of expertise	*GI/ST (only)*		*GI/ST (also)*			*Other*		*GI/ST (only)*		*GI/ST (also)*			*Other*	
Number of pathologists	9		28			2		12		55			4	
Years of activity Mean (min-max)	16.37 (1–36)							16.33 (1–35)						
Ocular field number*	**17**	**18**	**20**	**22**	**23**	**25**	**≥ 26**	**17**	**18**	**20**	**22**	**23**	**25**	**≥ 26**
Number of pathologists	0	2	8	14	3	5	6	2	1	20	39	3	4	3
	*Corresponding HPF*							*Corresponding area (mm*^*2*^*)*						
	35	31	25.5	21	19	17.5	15	7.1	8	9.8	11.9	13.1	14.2	16.6
Prognostic scheme	**A**	**B**	**C**	**D**	**E**	**F**	**G**	**A**	**B**	**C**	**D**	**E**	**F**	**G**
Number of pathologists	5	29	3	1	3	10	3	20	58	14	8	7	2	2

*GI*, gastrointestinal pathology; *ST*, soft tissue pathology; *A*, Fletcher et al. [1]; *B*, Miettinen et al. [2]; *C*, Joensuu [3]; *D*, Rossi et al. [4]; *E*, Joensuu et al. [6]; *F*, other prognostic schemes; *G*, none

^*^One pathologist evaluated MC with a digital microscope and digitally selected the 5 mm^2^ area and is therefore not accounted for

Data concerning tumour site, size and MC were than reported in the database, and risk class for each case was evaluated by applying the prognostic scheme by Miettinen [2].

### Statistical analysis

Comparisons between groups were explored using Student’s *t* test for paired samples. *P* was considered significant when ≤ 0.05.

## Results

### Survey

One hundred and ten pathologists participated in the survey. Data are summarized in Table 1. Pathologists were divided in 2 subgroups on the basis of the method used for counting mitoses: 5 mm^2^ (39 pathologists, 35.5%) vs 50 HPFs (71 pathologists, 64.5%). The two groups are homogenous concerning years of professional activity and area of expertise (*p*=0.435).

No significant differences were noted in microscope ocular FN and in prognostic scheme used. Only one pathologist uses a digital microscope for counting mitoses in digitally selected 5 mm^2^; in all the other cases, MC was performed at the microscope.

The prognostic scheme by Miettinen [2] was the most used (87/110 — 79%) followed by the Fletcher scheme [1] (25/100 — 23%), and Joensuu scheme [3] (17/110 — 15.5%). Of note, all these prognostic schemes evaluate MC as a categorical variable. On the other hand, prognostic schemes where MC is evaluated as a continuous variable [4, 5] were rarely used (8% and 9% respectively). Twelve pathologists used the prognostic schemes reported in WHO 2019 or CAP 2021, which both refer to the Miettinen scheme [2]. Seventy-seven pathologists used only one prognostic scheme, and in this case the most frequently applied is the Miettinen scheme [2] (59/77 — 76.6%) while 27 pathologists used 2 or more (up to 5) prognostic schemes. Only 5 pathologists used no prognostic scheme in their report. More interestingly, the majority of participants (64.5%) did not express MC in 5 mm^2^ but in 50 HPF, with variable counting areas greater than 5 mm^2^, from 7.1 mm^2^ for oculars with FN 17 to a maximum of 16.6 mm^2^ or more for oculars with FN 26–26.5 (Table 1). The majority of pathologists use oculars which have a FN of 20 or 22 (59/71 — 83%).

### Histologic evaluation

Mitoses counted in 5 mm^2^ versus 7.95 mm^2^ (corresponding to 50 HPFs with FN 18), 9.8 mm^2^ (corresponding to 50 HPFs with FN 20), and 11.85 mm^2^ (corresponding to 50 HPFs with FN 22) resulted in risk class change in 10/100 cases (10%), 28/100 (28%) cases and 41/100 (41%) cases respectively (Table 2). As expected, the number of cases with change in risk class increased consensually with the increase of evaluation area for GISTs with very low risk (VLR), low risk (LR) and moderate risk (MR), while no change was possible in already high-risk cases. Of note is that change in risk category is not necessarily to the next category. Indeed, in some cases, risk category changed from VLR to MR or from LR to HR.

**Table 2:** Variations in prognostic class assignment (according to Miettinen et al. [2]) when counting mitoses in 5 mm^2^ versus in 50 HPFs with ocular field numbers of varying sizes (18 to 22)

Prognostic scheme	MC/5 mm^2^	MC/50 HPF					
		FN 18–8 mm^2^		FN 20–9.8 mm^2^		FN 22–11.9 mm^2^	
Miettinen et al. [2]	25 VLR	23 VLR	2 MR	17 VLR	8 MR	13 VLR	12 MR
	25 LR	20 LR	5 HR	14 LR	11 HR	10 LR	15 HR
	25 MR	22 MR	3 HR	14 MR	9 HR	11 MR	14 HR
	25 HR	25 HR		25 HR		25 HR	

*MC*, mitotic count; *FN*, field number; *VLR*, very low risk; *LR*, low risk; *MR*, moderate risk; *HR*, high risk

## Discussion

The expansion of medical knowledge follows small steps, with sequential changes to how we interpret and diagnose diseases. However, any change requires diffusion to all operators and its integration into daily clinical practice. In this specific scenario, the change of how a pathologist must count mitoses may greatly influence risk stratification in GISTs.

Recent guidelines suggest that MC should be performed in 5 mm^2^ thus substituting the previous evaluation on 50 HPF, and the reason for this change is essentially related to the need for a standardized counting area. Despite this, our survey demonstrates that the majority of Italian pathologists still perform MC evaluation on 50 HPF, consequently counting mitoses in an area which is always greater than 5 mm^2^ and which can reach, with modern light microscopes mounting oculars with FN 26 or higher, more than 3 times the size (16.6 mm^2^) of the area which should be evaluated. This is not simply a consequence of the pathologist’s individual area of expertise. Indeed, pathologists with a special interest in gastrointestinal and/or soft tissue pathology are almost equally represented in both groups, as well as pathologists who are involved in gastrointestinal and/or soft tissue pathology, only as a part of their routine workload.

The most frequently adopted prognostic schemes [1–3] use MC as a categorical variable (cutoff value of ≤5 vs >5 mitoses) and this, coupled with a larger area of MC evaluation, favours a change of risk class which can potentially lead to inappropriate adjuvant therapy for patients. The second aim of this study was specifically to evaluate this aspect, using 100 GISTs (25 for each risk class of the Miettinen scheme). Our results show that a change in class risk was observed in a relevant number of cases (from 10% to 41%) and that this obviously correlated with the increasing dimensions of the measured area. This effect was seen in all the risk classes (VLR, LR, MR) except for HR. The increase of class risk for already categorized HR cases is not possible when using MC as a categorical variable, but it can rise, when expressed as percentage of relapse risk, with prognostic schemes [4, 5] using MC as a continuous variable.

Audit of real-world practice is an important step in understanding how pathology practice can lead to errors. In our survey, all the pathologists who count 50 HPFs (64.5%) with ocular FNs ranging between 17 and 26.5 are counting way too much area. This practice is not limited to routine pathology; indeed, discrepancies in area assessment can be found in research studies also. Nishida et al. [17] report on a central pathological review of high-risk GISTs and show some discrepancies in MC. Of interest is that the authors specify that MC during central revision was performed on 50 HPFs with a FN of 26.5 corresponding to an area of 17.2 mm^2^. So, if experienced pathologists fall foul to FN discrepancies, our results in the real world should not be too surprising.

Limitations to this study include a possible bias in survey participation, which was limited to Italian pathologists and may not completely represent worldwide daily practice. Indeed, it is possible that only pathologists with some interest in GIST diagnosis participated in the survey. A further limitation is that all cases were re-evaluated by the same pathologists and therefore interobserver variability, which is an important factor leading to MC discrepancies, could not be evaluated.

In conclusion, this study demonstrates that MC is still performed, in a great number of cases, on 50 HPFs rather than the guideline suggested 5 mm^2^. This can significantly influence the attribution of risk of relapse/metastases, as increase in ocular FN, and consequently of the measured area, leads to higher MCs.

## Data Availability

The datasets generated during and/or analysed during the current study are available from the corresponding author on reasonable request. The study was conducted according to the guidelines of the Declaration of Helsinki. Ethics committee approval was not required due to the following considerations: all data related to patients’ identification were anonymized; only original slides stained with haematoxylin and eosin were reassessed and no new sections were produced; this study has a speculative aim and results will not modify in any way the diagnosis and prognosis or add new clinical information useful for patient management.
